# Esophageal Pathology in Asymptomatic and Symptomatic Patients with Obesity Undergoing Evaluation for Bariatric Surgery

**DOI:** 10.1007/s11605-021-05169-w

**Published:** 2021-10-20

**Authors:** Priya Sharma, Fady Youssef, Madeline Greytak, Ryan Broderick, Garth Jacobsen, Santiago Horgan, Rena Yadlapati

**Affiliations:** 1 Department of Medicine, University of California, La Jolla, San Diego, CA USA; 2 Division of Gastroenterology, Center for Esophageal Diseases, University of California, ACTRI, Building 1W517, 9500 Gilman Drive MC 0956, La Jolla, San Diego, CA 92093 USA; 3 Department of Surgery, University of California, La Jolla, San Diego, CA USA; 4 Bariatric and Metabolic Institute, University of California, La Jolla, San Diego, CA USA

**Keywords:** Esophageal motility, Gastroesophageal reflux disease, Ambulatory reflux monitoring, High-resolution manometry, Bariatric surgery

## Introduction

Bariatric surgery is the gold standard in achieving long-term weight loss [1]. Esophageal pathology, including gastroesophageal reflux disease (GERD) and esophageal dysmotility, is common after bariatric surgery. However, it is often unclear as to whether pathology exists in obesity prior to surgery. Both anatomic and physiologic processes in obesity can impact esophageal function, which in turn may impact outcomes following surgery [2–5]. Overall, the prevalence and characteristics of esophageal disorders in obesity are not well understood.

Further, the value of esophageal physiologic testing prior to bariatric surgery, such as barium esophagram, reflux monitoring, or high-resolution manometry (HRM), is not well defined or standardized across centers. At our center, the standard protocol requires pre-operative esophageal HRM (Medtronic, Minneapolis, MN) for all patients, whether symptomatic or asymptomatic, and often includes esophagogastroduodenoscopy, ambulatory reflux monitoring, and barium esophagram. Thus, the primary aims of this study were to characterize esophageal physiology in patients with obesity and compare physiologic patterns between patients with and without esophageal symptoms. Based on these findings, we hypothesize our findings will support the utility of HRM prior to bariatric surgery, particularly in patients with symptoms.

## Methods

This retrospective study included adult patients with obesity (body mass index (BMI) ≥ 35 kg/m^2^) undergoing preoperative bariatric surgical evaluation at a single tertiary care center between 2/2019 and 2/2020. Symptoms were recorded based on routine standardized patient-reported instruments. Asymptomatic patients were those without dysphagia, heartburn, regurgitation, or non-cardiac chest pain. Symptomatic presentation was defined as reporting at least one of these symptoms. Motility diagnosis was determined per Chicago Classification version 3.0 [6]. Objective GERD was defined as meeting one of the following criteria: (1) acid exposure time (AET) ≥ 4.0%, (2) presence of erosive esophagitis on endoscopy, or (3) confirmed Barrett’s esophagus. Primary surgical data collected included the type of weight loss surgery performed: Roux-en-Y gastric bypass (RYGB) or laparoscopic sleeve gastrectomy.

## Results

A total of 300 adult patients underwent bariatric surgical evaluation with HRM and were included in this analysis: mean age 46.3 ± 13.6 years, 226 (75.3%) female, mean BMI 45.2 ± 8.8 kg/m^2^. Of the 300, 196 (65.3%) were symptomatic and 104 (34.7%) were asymptomatic (Table 1). Symptom presentation was as follows: 56.3% heartburn, 28.7% dysphagia, 27.0% regurgitation, and 29.3% noncardiac chest pain.

**Table 1:** Baseline characteristics, manometric findings, and objective GERD among asymptomatic and symptomatic patients

Variable	Asymptomatic (*n* = 104)	Symptomatic (*n* = 196)	*p*-value
Age, years	43.0 ± 13.1	48.1 ± 13.5	0.002
Female gender	79 (76%)	147 (75%)	0.85
BMI, kg/m^2^	47.7 ± 8.5	43.9 ± 8.7	< 0.001
*High-resolution manometry*			
Hiatal hernia	23 (34%)	61 (38%)	0.64
Hiatal hernia size, cm	0.40 ± 1.11	0.62 ± 1.42	0.18
EGJ baseline pressure, mmHg	31.3 ± 13.8	27.8 ± 14.5	0.04
Median IRP, mmHg	10.69 ± 6.2	10.04 ± 7.9	0.47
Mean DCI, mmHg-s-cm	2257 ± 1708	1825 ± 1398	0.02
Mean distal latency, s	6.8 ± 1.6	7.0 ± 2.4	0.42
% bolus clearance incomplete	21 ± 29	22 ± 32	0.67
*Objective GERD*			
Esophageal acid exposure time (% time pH < 4.0)	6.8 ± 1.6 (*n* = 4)	7.6 ± 7.1 (*n* = 63)	0.82
Esophageal acid exposure time > 4.0%	2 (1.9%)	40 (20.4%)	< 0.001
Erosive esophagitis	1 (1.4%) (*n* = 73)	19 (11.3%) (*n* = 168)	
Barrett’s esophagus	3 (4.1%) (*n* = 73)	11 (6.5%) (*n* = 168)	
Objective GERD (defined as: AET > 4.0%, erosive esophagitis, and/or Barrett’s esophagus)	5 (6.8%) (*n* = 73)	55 (32.4%) (*n* = 170)	< 0.001

EGJ, esophagogastric junction; IRP, integrated relaxation pressure; DCI, distal contractile integral; GERD, gastroesophageal reflux disease; AET, acid exposure time. Continuous data presented as mean ± standard deviation, categorical data presented as *n* (%)

**Fig. 1:**
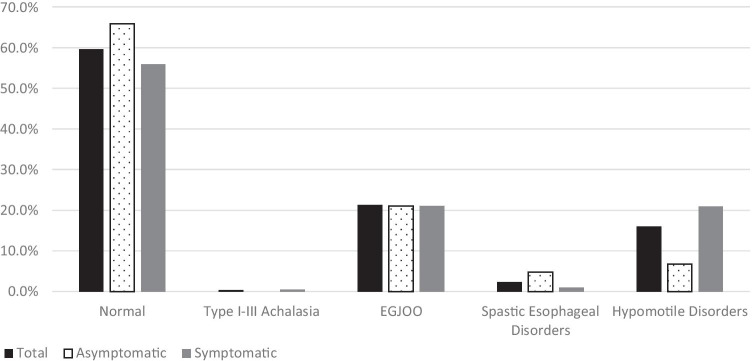
Distribution of motility disorders between total, asymptomatic, and symptomatic patients. Esophagogastric junction outflow obstruction (EGJOO); spastic esophageal disorders include distal esophageal spasm (DES) and hypercontractile esophagus; hypomotile esophageal disorders include ineffective esophageal motility (IEM), fragmented peristalsis, and absent contractility

On HRM, abnormal esophageal motility was seen in 40.3% of all patients, with 34% of asymptomatic patients having abnormal motility patterns. Distribution of esophageal motility disorders significantly differed with a greater proportion of ineffective esophageal motility in symptomatic vs asymptomatic patients (36 (18%) vs 7 (7%); *p* = 0.03). The distribution of manometric esophagogastric junction outflow obstruction was similar among symptomatic and asymptomatic patients (21% vs 21%) (Fig. 1). Baseline esophago-gastric junction pressure was higher among symptomatic vs asymptomatic patients asymptomatic vs symptomatic (27.8 ± 14.5 vs 31.3 ± 13.8 mmHg*, p* = 0.04).

Ability to assess for objective GERD was available for 243 (81%) patients: 170 (70.0%) symptomatic and 73 (30.0%) asymptomatic. Among the 243 patients, 32.4% of symptomatic vs 6.8% of asymptomatic patients had evidence of objective GERD, providing 5.3 times higher odds of objective GERD in symptomatic patients compared to asymptomatic patients (95% CI 1.45, 20.0; *p* = 0.01). Of those who underwent bariatric surgery, symptomatic patients were more likely to receive RYGB compared to asymptomatic patients (33% vs 7%) and less likely to receive sleeve gastrectomy (67% vs 93%; *p* < 0.01).

## Conclusion

Esophageal dysmotility and reflux are common in obesity. Symptoms of heartburn, regurgitation, dysphagia, and non-cardiac chest pain may suggest abnormal esophageal motility and/or esophageal reflux. These data suggest a role of HRM and reflux monitoring in patients with esophageal symptoms including obstructive and typical reflux symptoms prior to bariatric surgery, as detection of dysmotility and objective GERD may influence preoperative course.

## Abbreviations

GERD: Gastroesophageal reflux disease
HRM: High-resolution manometry
BMI: Body mass index
AET: Acid exposure time
RYGB: Roux-en-Y gastric bypass
